# Neurodevelopmental predictors of treatment response in schizophrenia and bipolar disorder

**DOI:** 10.1017/S0033291724001776

**Published:** 2024-10

**Authors:** Anton Iftimovici, Emma Krebs, William Dalfin, Adrien Legrand, Linda Scoriels, Gilles Martinez, Narjes Bendjemaa, Edouard Duchesnay, Boris Chaumette, Marie-Odile Krebs

**Affiliations:** 1 Université Paris Cité, Institute of Psychiatry and Neuroscience of Paris (IPNP), INSERM U1266, Paris, France; 2 Institut de Psychiatrie, CNRS GDR 3557, Paris, France; 3 GHU Paris Psychiatrie et Neurosciences, Paris, France; 4 NeuroSpin, Atomic Energy Commission, Gif-sur Yvette, France; 5 Department of Psychiatry, McGill University, Montréal, Québec, Canada

**Keywords:** schizophrenia, bipolar disorder, neurological soft signs, neurodevelopment, age at onset of disease, treatment response

## Abstract

**Background:**

Treatment resistance is a major challenge in psychiatric disorders. Early detection of potential future resistance would improve prognosis by reducing the delay to appropriate treatment adjustment and recovery. Here, we sought to determine whether neurodevelopmental markers can predict therapeutic response.

**Methods:**

Healthy controls (*N* = 236), patients with schizophrenia (*N* = 280) or bipolar disorder (*N* = 78) with a known therapeutic outcome, were retrospectively included. Age, sex, education, early developmental abnormalities (obstetric complications, height, weight, and head circumference at birth, hyperactivity, dyslexia, epilepsy, enuresis, encopresis), neurological soft signs (NSS), and ages at first subjective impairment, clinical symptoms, treatment, and hospitalization, were recorded. A supervised algorithm leveraged NSS and age at first clinical signs to classify between resistance and response in schizophrenia.

**Results:**

Developmental abnormalities were more frequent in schizophrenia and bipolar disorder than in controls. NSS significantly differed between controls, responsive, and resistant participants with schizophrenia (5.5 ± 3.0, 7.0 ± 4.0, 15.0 ± 6.0 respectively, *p* = 3 × 10^−10^) and bipolar disorder (5.5 ± 3.0, 8.3 ± 3.0, 12.5 ± 6.0 respectively, *p* < 1 × 10^−10^). In schizophrenia, but not in bipolar disorder, age at first subjective impairment was three years lower, and age at first clinical signs two years lower, in resistant than responsive subjects (*p* = 2 × 10^−4^ and *p* = 9 × 10^−3^, respectively). Age at first clinical signs and NSS accurately predicted treatment response in schizophrenia (area-under-curve: 77 ± 8%, *p* = 1 × 10^−14^).

**Conclusions:**

Neurodevelopmental features such as NSS and age of clinical onset provide a means to identify patients who may require rapid treatment adaptation.

## Introduction

Treatment resistance in psychiatric disorders constitutes a major public health challenge. Its prevalence has been estimated at between 20 and 60% of individuals with schizophrenia (Howes, Thase, & Pillinger, 2022). It impacts quality of life in all its physical, psychological, and social domains (Iasevoli et al., 2016; Lex et al., 2019). In bipolar disorder, the exact prevalence of resistance is unknown, but the lack of response to a first-line treatment may exceed 40% and is associated with increased comorbidity and suicide risk (Fornaro et al., 2020; Yatham et al., 2018). Yet, despite its high socio-economic burden, treatment resistance remains under-investigated. While there is a consensus on the fact that two lines of treatment should have been tried to speak about resistance, it is challenging to evaluate, as it requires certainty of the underlying disease, of adequate treatment (the right treatment, at the right dosage, for a sufficient amount of time), and a clear assessment of non-response (Howes et al., 2022). Hence, although in some conditions there is a recommended medication for treatment resistance, such as clozapine in schizophrenia, it is widely underused (Bachmann et al., 2017), which highlights the delay in proper diagnosis of treatment-refractory diseases (Yasui-Furukori et al., 2022). Better characterization of the clinical correlates of initial treatment resistance or response is needed to improve care, by decreasing the delay before an adaptation of therapeutic strategy is proposed in patients at risk of resistance. Several reports indicate that treatment resistance is, at least partly, reflecting an intrinsic feature of a subgroup of patients with schizophrenia, present since the first episode (Lally et al., 2016; O'Donoghue, Mora, Bismark, Thompson, & McGorry, 2024; Wong et al., 2024). Furthermore, as cortical gyrification could help identify patients at high-risk of treatment resistance, it would suggest that treatment response could be related to specific developmental trajectories (Ajnakina et al., 2021).

In schizophrenia, the strongest predictors of therapeutic resistance are a younger age at onset and poor premorbid social functioning (Legge et al., 2020). Early onset before 18 is associated with increased refractoriness to antipsychotics (Iasevoli et al., 2022), which is also in favor of a neurodevelopmental determinant in this resistant subtype (Chan et al., 2021). According to the neurodevelopmental paradigm, the emergence of psychiatric diseases depends on the timing of disruption in brain maturation, with early alterations leading to severe developmental phenotypes such as autism spectrum disorder, while later alterations may drive the phenotypes toward schizophrenia (Casey, Oliveri, & Insel, 2014). Age at onset of the first episode thus appears as a clinical cursor on the neurodevelopmental timeline (Marín, 2016), albeit the onset may be revealed by less specific symptoms, before the onset of psychotic symptoms classically used to determine schizophrenia onset. The link between early age of onset and neurodevelopment is further supported by the shared burden of deleterious copy number variants between early-onset psychosis and autism spectrum disorders (Brownstein et al., 2022), and the phenotypic continuum between autism and schizophrenia in young subjects (Martinez et al., 2017), in particular for childhood- or very early-onset schizophrenia (SCZ) (Driver, 2020). This may also be the case for bipolar disorder, where there is increasing evidence for a neurodevelopmental dimension (Craddock & Owen, 2010) influencing response to treatment : early age of onset of bipolar disease was associated with greater severity (Joslyn, Hawes, Hunt, & Mitchell, 2016), lower rates of recovery (Treuer & Tohen, 2010) as well as with abnormal neurodevelopmental trajectories (Corponi et al., 2023).

However, despite the potential relevance of the neurodevelopmental dimension for disease outcome, age at onset is often the only considered variable, especially in schizophrenia (Smart, Kępińska, Murray, & MacCabe, 2021). Other neurodevelopmental markers, such as a history of developmental delays or the presence of neurological soft signs (NSS) could also be crucial to predict this outcome. NSS comprise subtle sensorimotor abnormalities that reflect brain immaturity and whose persistence into adulthood is considered a marker of neurodevelopmental burden (D'Agati, Pitzianti, Curatolo, & Pasini, 2018). Their presence has been linked to treatment resistance in schizophrenia (de Bartolomeis et al., 2018). For this reason, and in view of the biological continuum between psychiatric disorders, the use of such neurodevelopmental markers to identify subtypes with a common underlying vulnerability may be highly relevant to predict treatment resistance transdiagnostically (Howes et al., 2022). Moreover, since these markers are available from early, clinically subthreshold stages of disease evolution, they can be operationalized as part of a transdiagnostic clinical staging approach (Shah et al., 2020). Integrating the assessment of potential treatment resistance in the staging framework would allow for more informed early intervention and closer clinical and therapeutic monitoring of the subset of patients most at risk of treatment resistance, thus reducing the delay to recovery.

In this work, we hypothesize that the presence of neurodevelopmental markers, such as NSS and age at disease onset, could help distinguish among subjects with schizophrenia or bipolar disorder those who have an excellent immediate response in the first six months from those who will not. This will allow for early detection of individuals who will require a rapid therapeutic adjustment.

## Methods

### Sample

Participants were drawn from the PSYDEV transdiagnostic cohort ('Familial and Genetic Study of Developmental Aspects of Psychiatric Illness', PI MO Krebs, Sponsor Inserm) (Girard et al., 2011). Diagnoses of schizophrenia and bipolar disorder were made according to the DSM-IV criteria, using semi standardized interviews (DIGS 3.0) by trained clinicians. We included in the analysis all subjects with schizophrenia or bipolar disease with a known treatment response (*N* = 280 and *N* = 78, respectively) and healthy controls (*N* = 236) with a follow up of at least 6 months. The cohort received all ethical and regulatory approvals (Comité de Protection des Personnes, Ile-de-France IV), and informed written consent was obtained from each subject or their legal representatives.

### Measures

Initial response to treatment was characterized with the use of May & al. scale (Brenner et al., 1990), clinical assessment, as well as follow-up information for at least six months. Resistance was assessed as insufficient or no recovery as a whole, and not only on the persistence of symptoms.

In schizophrenia, treatment responsive patients were defined as very good responders to antipsychotics with a complete response in less than three months following May & Dencker criteria (⩽ 3) during the initial phase of treatment and who remained responders in the six months follow-up. Treatment resistant patients were defined as all individuals who did not meet the above criteria for good response: with either only partial initial response (⩾ 4) and/or who became partial or bad responders in the follow up, including those who were later prescribed clozapine.

In bipolar disease, according to the Canadian Network for Mood and Anxiety Treatments, resistance was considered if a first-line of mood stabilizer (lithium) was not sufficient after two weeks for manic patients, and one month for bipolar depression (two months if lamotrigine was used, given the necessary slow titration) (Yatham et al., 2018). When available, the Alda scale was used for lithium response, with a threshold of response superior or equal to seven.

For all patients, assessments were performed with direct interviews, including semi-standardized interviews for lifetime diagnosis and developmental, clinical therapeutic and family history (DIGS 3.0), and validated scales : GAF and BPRS (for schizophrenia and bipolar disorder), PANSS (for schizophrenia), YMRS and MADRS (for bipolar disorder). In addition, we also used all information available at the time of interview, from the family, the practitioners and case report files. It should be noted that the patients were engaged in clinical care and thanks to the organization of mental health in France in catchment areas, several years of prospective follow-up were often available.

Socio-demographic characteristics included age, sex, and education. Developmental history included the presence of obstetric complications, birth height, weight, head circumference, the presence of hyperactivity, dyslexia, epilepsy, enuresis, and encopresis. These variables were selected because they were the ones adult patients and their families could most reliably recall and/or were written in their child health record. Psychiatric history included the presence of a diagnosis of schizophrenia or mood disorder in a first or second degree relative, as well as ages at first subjective impairment, first clinical signs, first treatment, and first hospitalization. Age at first subjective impairment was assessed by asking the question: ‘for you, when did your difficulties actually begin ?’. Age at first clinical signs was the age when the first objective symptoms or signs of a major psychiatric syndrome appeared.

NSS examination comprised 23 items divided in three factors (motor integration, motor coordination, and sensory integration) investigating gait and balance, coordination precision on various praxis tasks, speed and dysrhythmia in rapid alternating movements, stereognosis and graphesthesia, lateral preference and right/left discrimination. For every item, rating ranges from 0 to 3, with an explicit definition and descriptive anchors. This scale showed good internal consistency (Cronbach's *α* = 0.85) and inter-rater reliability (score of 0.82) in a separate study (Krebs, Gut-Fayand, Bourdel, Dischamp, & Olié, 2000). For a subgroup of patients we had both a first NSS examination in antipsychotic naïve or untreated individuals and a later assessment of treatment response (bipolar disease *N* = 23, or schizophrenia, *N* = 21). In addition, NSS examination included the Simpson-Angus scale, assessing the neurological side-effects of antipsychotics.

### Statistical analysis

For each variable common to control, treatment response, and treatment resistance groups, we applied one-way ANOVA tests or χ^2^ for three groups. Then three pairwise post-hoc Tukey tests were done for each variable. *t* tests or χ^2^ for two groups were used for comparisons of variables only relevant for comparison between treatment response and resistance groups. We corrected for multiple testing using the false discovery rate. For computation and illustration of effect-sizes of the significant variables of interest (NSS and age at first clinical signs), the Data Analysis with Bootstrap-coupled Estimation package was used to build bootstrap 95% confidence intervals of the effect-size, computed with Cohen's d (Ho, Tumkaya, Aryal, Choi, & Claridge-Chang, 2019).

### Machine-learning analysis

In a five-fold cross-validation loop, we fitted the response status (resistant or responsive) on the significant variables (three factors of NSS and age at first clinical signs) with a logistic regression model in each training set. The classifier was a logistic regression with: (i) a norm L2 penalty, chosen because it outputs weights for each variable, allowing interpretation of each weighted variable relative to the others, (ii) a nested cross-validation with hyperparameter set inside the training loop to *C* = 0.1, to have only a limited regularization in this context of observations outnumbering the variables, leading to a convergence toward one single solution, and (iii) a class weight set to ‘balanced’ to account for the higher frequency of treatment-resistant subjects. The preprocessing step of residualizing for age and sex was nested inside the cross-validation.

We then used the model for prediction in each corresponding testing set, with the scikit-learn package (Pedregosa et al., 2018). Average prediction performance was assessed across the five folds with the area-under-the-curve (AUC), F1-score, specificity, sensitivity, and positive predictive value. We then computed the *p* value associated with the classification rate, by comparing the number of correct classifications to the null hypothesis of binomial distribution of parameters, with a 50% chance level for 197 subjects with available age at first onset of clinical signs.

## Results

### NSS between controls, subjects with schizophrenia and with bipolar disorder

**Figure 1.**
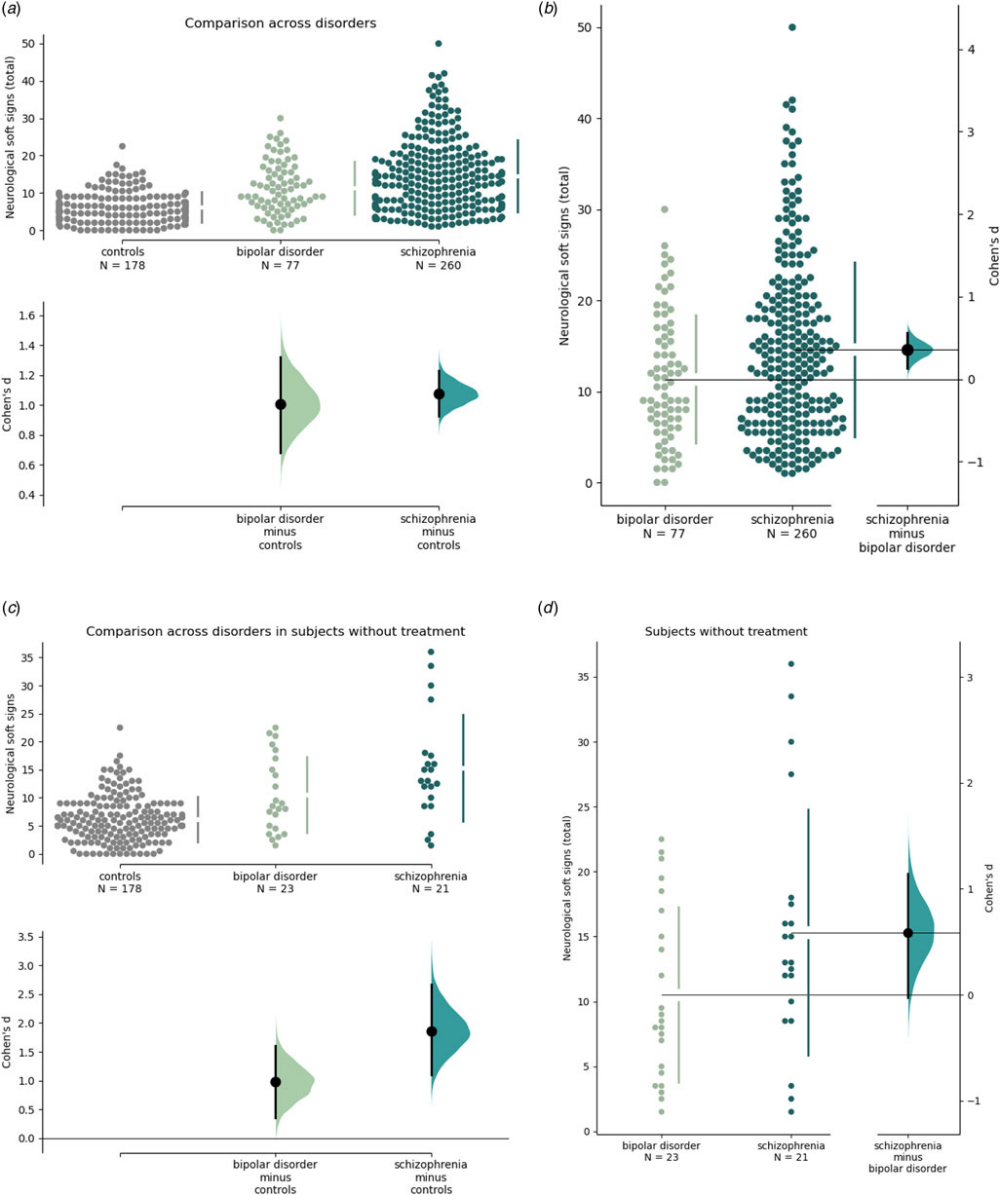
Effect-size of the difference in neurological soft signs between healthy controls, schizophrenia, and bipolar disorder. Bootstrapped 95% confidence interval of the effect-size between: (a) controls and schizophrenia or bipolar disorder (*d* = 1.13, *p* < 1 × 10^−10^ and *d* = 1.00, FDR-adjusted *p* < 1 × 10^−10^ respectively), (b) schizophrenia and bipolar disorder (*d* = 0.35, FDR-adjusted *p* = 0.006), (c) controls and schizophrenia or bipolar disorder without treatment (*d* = 1.88, *p* < 1 × 10^−10^ and *d* = 0.98, *p* = 2 × 10^−5^ respectively), and (d) schizophrenia and bipolar disorder without treatment (*d* = 0.56, *p* = 0.06).

NSS were higher in subjects with schizophrenia and bipolar disorder than controls (*d* = 1.13, *p* < 1 × 10^−10^ and *d* = 1.00, *p* < 1 × 10^−10^ respectively, Fig. 1a), and higher in schizophrenia than in bipolar disorder (*d* = 0.35, *p* = 0.006, Fig. 1b). NSS were also higher than controls when considering subjects without treatment only, both in schizophrenia and bipolar disorder (*d* = 1.88, *p* < 1 × 10^−10^ and *d* = 0.98, *p* = 2 × 10^−5^ respectively, Fig. 1c), and higher in schizophrenia than in bipolar disorder, although not significantly (*d* = 0.56, *p* = 0.06, Fig. 1d). NSS were positively correlated with extrapyramidal side-effects (*r* = 0.31, *p* = 1 × 10^−6^), but there was no difference in extra-pyramidal side-effects between response and resistance to treatment (Table 1).

**Table 1.** Differences between healthy controls, treatment responsive schizophrenia, and treatment resistant schizophrenia

	Groups			Tests		Post-hoc Tukey tests (*p* value)		
	Controls (*N*)	Schizophrenia treatment response (*N*)	Schizophrenia treatment resistance (*N*)	Anova F or χ^2^ (for 3 groups) T or χ^2^ (for 2 groups)	FDR *p* value	Treatment response v. Controls	Treatment resistance v. Controls	Treatment response v. resistance
Socio-demographic characteristics								
Age – years	24 ± 4 (236)	27.5 ± 5.5 (94)	29 ± 6 (186)	13.44	4 × 10^−6^	0.04	1 × 10^−6^	0.25
Sex – F/M	103 / 133	20 / 74	61 / 125	15.7	6 × 10^−4^	2 × 10^−4^	0.03	0.06
Education – years of study	14 ± 2 (200)	12 ± 2 (92)	11.5 ± 2 (182)	32.3	3 × 10^−10^	3 × 10^−4^	< 1 × 10^−10^	0.03
Familial history								
Familial history of psychosis – %	–	31 (94)	39 (186)	2.03	0.18	–	–	–
Familial history of mood disorder – %	–	36 (94)	42 (186)	0.06	0.84	–	–	–
Developmental history								
Obstetric complications – %	8 (213)	22 (74)	25 (149)	33.7	1 × 10^−7^	3 × 10^−5^	5 × 10^−8^	0.80
Birth height – cm	50 ± 2 (88)	50 ± 1 (28)	50 ± 1 (54)	0.15	0.86	0.90	0.90	1.00
Birth weight – kg	3.3 ± 0.3 (104)	3.3 ± 0.5 (43)	3.3 ± 0.3 (86)	0.51	0.64	0.62	0.77	0.93
Head circumference – cm	35 ± 1 (52)	34 ± 1 (15)	35 ± 1 (21)	0.60	0.61	1.00	0.56	0.66
Hyperactivity – %	1 (73)	13 (78)	8 (155)	7.26	0.04	0.02	0.14	0.22
Dyslexia – %	3 (195)	17 (87)	13 (173)	18.0	2 × 10^−4^	7 × 10^−5^	8 × 10^−4^	0.44
Epilepsy – %	0 (196)	2 (85)	5 (172)	12.0	3 × 10^−3^	0.17	2 × 10^−3^	0.36
Enuresis – %	2 (229)	20 (89)	18 (178)	39.5	8 × 10^−9^	6 × 10^−9^	1 × 10^−8^	0.73
Encopresis – %	0 (195)	6 (88)	4 (176)	11.7	5 × 10^−3^	1 × 10^−3^	0.01	0.51
Psychiatric history								
First subjective symptoms – years	–	19 ± 3 (83)	16 ± 4 (168)	−3.90	**2** × 10^−4^	–	–	–
First clinical signs – years	–	19 ± 3 (68)	17 ± 3 (145)	−2.76	**9** × 10^−3^	–	–	–
First treatment – years	–	22 ± 3 (78)	20 ± 3 (169)	−2.55	**0.01**	–	–	–
Hospitalization – %	–	89 (89)	91 (176)	2.36	0.15	–	–	–
First hospitalization – years	–	22 ± 3 (78)	21 ± 4 (166)	−0.76	0.52	–	–	–
Clinical features								
Global assessment of functioning	–	50 ± 10 (42)	40 ± 10 (113)	−6.20	**8** × 10^−9^	–	–	–
BPRS (18 items)	–	35 ± 10 (61)	48 ± 9 (152)	6.68	**3** × 10^−10^	–	–	–
PANSS total	–	60 ± 19 (61)	89 ± 15 (154)	8.05	**6** × 10^−13^	–	–	–
PANSS positive	–	13 ± 18 (61)	18 ± 5 (154)	4.53	**2** × 10^−5^	–	–	–
PANSS negative	–	17 ± 6 (61)	26 ± 5 (154)	7.96	**6** × 10^−13^	–	–	–
PANSS disorganization	–	7 ± 2 (61)	11 ± 2 (154)	6.72	**3** × 10^−10^	–	–	–
PANSS general psychopathology	–	32 ± 10 (61)	45 ± 9 (154)	7.34	**3** × 10^−11^	–	–	–
Extrapyramidal side effects (SAS)	–	9.0 ± 5 (94)	9.5 ± 4.5 (186)	0.85	0.39			
Neurological soft signs								
Total score	5.5 ± 3 (178)	7 ± 4 (87)	15 ± 6 (173)	99.6	3 × 10^−10^	1 × 10^−3^	< 1 × 10^−10^	**< 1** × 10^−10^
Motor coordination score	3.4 ± 2 (178)	5.0 ± 3 (87)	8.4 ± 4 (173)	57.3	3 × 10^−10^	0.02	< 1 × 10^−10^	**2** × 10^−8^
Motor integration score	1.4 ± 1 (178)	2.5 ± 2 (87)	5.0 ± 2 (173)	96.1	3 × 10^−10^	3 × 10^−3^	< 1 × 10^−10^	**< 1** × 10^−10^
Sensory integration score	1.3 ± 1 (178)	2.2 ± 1 (87)	3.7 ± 2 (173)	54.0	3 × 10^−10^	4 × 10^−3^	< 1 × 10^−10^	**8** × 10^−7^

*N*, number of subjects. Significant *p* values are computed after false discovery rate correction (Benjamini/Hochberg). Significant *p* values specific to the difference between resistance and response are in bold and light red. SAS, Simpson-Angus scale; BPRS, Brief Psychiatric Rating Scale; PANSS, Positive and Negative Syndrome Scale.

### Differences between control, responsive and resistant subjects with schizophrenia

**Figure 2.**
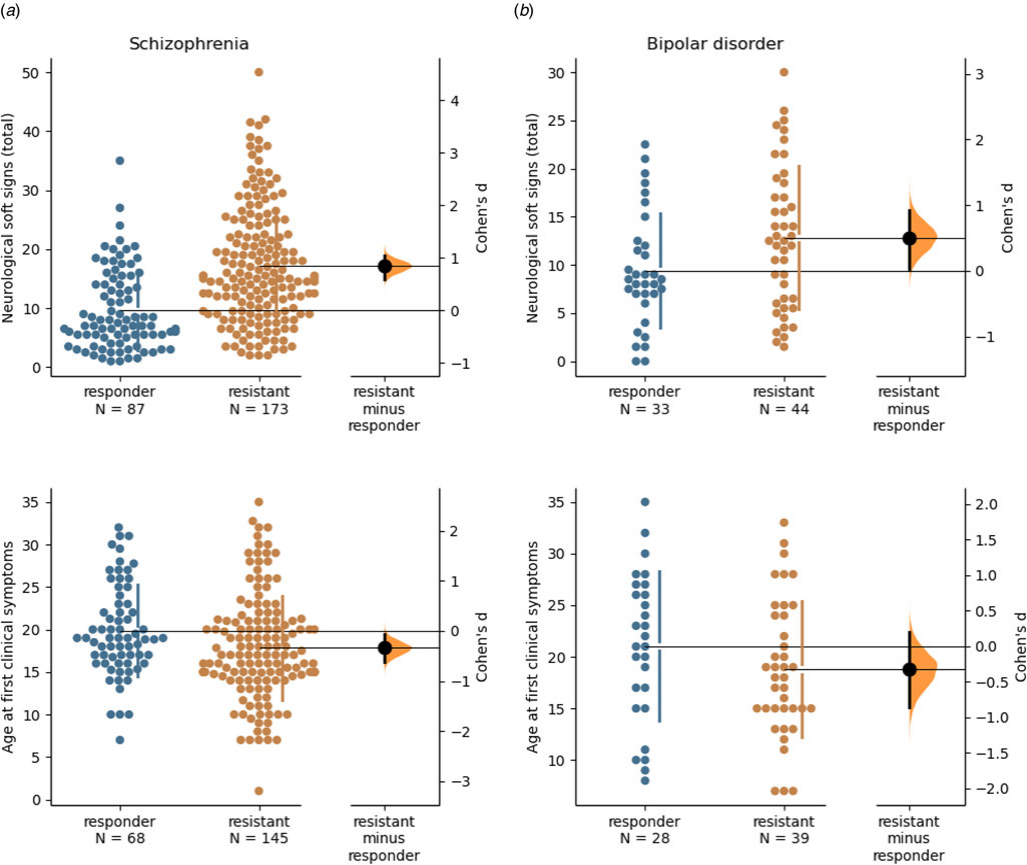
Bootstrapped 95% confidence interval of the effect-size of the difference for neurological soft signs and age at first clinical signs in (a) schizophrenia (*d* = 0.82, *p* < 1 × 10^−10^ and *d* = −0.35, *p* = 9 × 10^−3^ respectively), and (b) in bipolar disorder (*d* = 0.48, *p* = 0.01 and *d* = −0.3, *p* = 0.20, respectively).

As described in Table 1, patients with schizophrenia were older than controls, but there were no differences between responsive and resistant subjects regarding age at inclusion. There were more males among patients than in controls. Patients had fewer years of study than controls, and resistant subjects had slightly lower years of study than responsive. Treatment resistant subjects had overall more severe clinical symptomatology and functioning than treatment-responsive ones. In terms of developmental anomalies, there were more obstetric complications, epilepsy, dyslexia, and primary enuresis and encopresis in schizophrenia compared to controls, but there was no difference between responsive and resistant groups. In schizophrenia, NSS were significantly higher in treatment resistant than responsive patients (*d* = 0.82, *p* < 1 × 10^−10^, Fig. 2a). Compared to treatment responsive patients, first subjective symptoms (*d* = −0.45, *p* = 2 × 10^−4^), first clinical symptoms (*d* = −0.35, *p* = 9 × 10^−3^, Fig. 2a) and first treatment (*d* = −0.3, *p* = 0.01) occurred earlier in treatment resistant patients.

### Differences between control, responsive and resistant subjects with bipolar disorder

As described in Table 2, patients with bipolar disorder were older than controls, but there were no age differences between responsive and resistant subjects. Treatment-resistant subjects showed lower functioning and a higher clinical burden, with more depression, than treatment responsive ones. There were no differences in sex ratio or education. In terms of developmental anomalies, there were more hyperactivity, epilepsy, dyslexia, and primary enuresis in bipolar disorder compared to controls, but there was no difference between responsive and resistant groups. In bipolar disorder, NSS were significantly higher in treatment resistant than in responsive patients (*d* = 0.48, *p* = 0.01, Fig. 2b). Ages at onset were not different between the groups.

**Table 2.** Differences between healthy controls, treatment responsive bipolar disorder, and treatment resistant bipolar disorder

	Groups			Tests		Post-hoc Tukey tests		
	Controls (*N*)	Bipolar disorder treatment response (*N*)	Bipolar disorder treatment resistance (*N*)	Anova F or χ^2^ (for 3 groups) T or χ^2^ (for 2 groups)	FDR *p* value	Treatment response v. Controls	Treatment resistance v. Controls	Treatment response v. resistance
Socio-demographic characteristics								
Age – years	24 ± 4 (236)	38.5 ± 8.5 (34)	43 ± 7 (44)	81.0	< 1 × 10^−10^	< 1 × 10^−10^	< 1 × 10^−10^	0.62
Sex – F/M	103 / 133	18 / 16	26 / 18	4.13	0.13	0.40	0.08	0.75
Education – years of study	14 ± 2 (200)	12 ± 2 (33)	12 ± 2 (43)	0.35	0.70	0.93	0.77	0.71
Familial history								
Familial history of psychosis – %	–	24 (34)	30 (44)	0.11	0.74	–	–	–
Familial history of mood disorder – %	–	79 (34)	80 (44)	0.00	1.00	–	–	–
Developmental history								
Obstetric complications – %	8 (213)	12 (31)	20 (37)	8.21	0.02	0.64	0.01	0.38
Birth height – cm	50 ± 2 (88)	50 ± 0 (7)	50 ± 1.4 (12)	0.96	0.39	0.40	0.82	0.77
Birth weight – kg	3.3 ± 0.3 (104)	3.6 ± 0.2 (16)	3.3 ± 0.4 (22)	2.09	0.13	0.11	0.94	0.32
Head circumference – cm	35 ± 1 (52)	35 ± 0 (2)	33.8 ± 1.1 (6)	0.17	0.85	0.89	0.95	0.83
Hyperactivity – %	1 (73)	18 (33)	23 (44)	11.9	3 × 10^−33^	0.02	2 × 10^−3^	0.84
Dyslexia – %	3 (195)	18 (34)	18 (44)	16.2	3 × 10^−4^	4 × 10^−3^	1 × 10^−3^	1.00
Epilepsy – %	0 (196)	0 (34)	7 (44)	15.8	3 × 10^−4^	1.00	3 × 10^−3^	0.34
Enuresis – %	2 (229)	9 (34)	18 (44)	22.7	1 × 10^−5^	0.07	7 × 10^−6^	0.40
Encopresis – %	0 (195)	0 (34)	0 (44)	0.00	1.00	1.00	1.00	1.00
Psychiatric history								
First subjective symptoms – years	–	18 ± 7 (31)	17 ± 5 (42)	−0.97	0.33	–	–	–
First clinical signs – years	–	21.5 ± 5 (28)	18 ± 4 (39)	−1.30	0.20	–	–	–
First treatment – years	–	24.5 ± 4.5 (32)	23.5 ± 4 (42)	−0.59	0.56	–	–	–
Hospitalization – %	–	85 (34)	95 (44)	1.34	0.25	–	–	–
First hospitalization – years	–	26 ± 5 (29)	27 ± 7 (39)	0.39	0.70	–	–	–
Clinical features								
Global assessment of functioning	–	60 ± 10 (26)	55 ± 5 (31)	−3.42	**2** × 10^−3^	–	–	–
BPRS (18 items)	–	34 ± 7 (23)	47 ± 8 (32)	3.90	**3** × 10^−4^	–	–	–
YMRS	–	0 ± 0 (18)	2 ± 2 (26)	−0.30	0.77	–	–	–
MADRS	–	13 ± 11 (20)	22.5 ± 10 (34)	2.54	**0.01**	–	–	–
Neurological soft signs								
Total score	5.5 ± 3 (178)	8.5 ± 3 (33)	12.5 ± 6 (44)	32.5	< 1 × 10^−10^	3 × 10^−3^	< 1 × 10^−10^	**0.01**
Motor coordination score	3.4 ± 2 (178)	4.1 ± 2.5 (33)	6.0 ± 3 (44)	10.7	3 × 10^−5^	0.53	2 × 10^−5^	**0.04**
Motor integration score	1.4 ± 1 (178)	3.0 ± 2 (33)	4.0 ± 2 (44)	37.7	< 1 × 10^−10^	1 × 10^−4^	< 1 × 10^−10^	**0.03**
Sensory integration score	1.3 ± 1 (178)	2.4 ± 1.5 (33)	2.8 ± 1.5 (44)	18.0	5 × 10^−8^	2 × 10^−3^	3 × 10^−7^	0.44

*N*, number of subjects. Significant *p* values are computed after false discovery rate correction (Benjamini/Hochberg). Significant *p* values specific to the difference between resistance and response are in bold and light red. BPRS, Brief Psychiatric Rating Scale; YMRS, Young Mania Rating Scale; MADRS, Montgomery-Åsberg Depression Rating Scale.

### Treatment response prediction in schizophrenia

**Figure 3.**
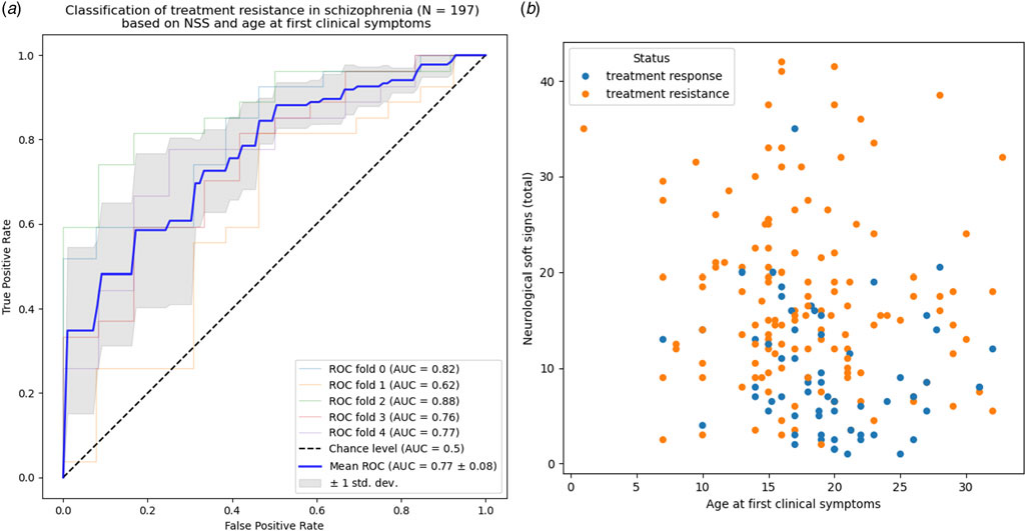
Prediction of treatment response based on age at first subjective symptoms and the three domains of neurological soft signs: motor coordination, motor integration, and sensory integration. (a) ROC curve and area-under-the-curve (AUC). (b) Distribution of responsive and resistant subjects with schizophrenia depending on NSS total (sum of the 3 dimensions) and age at onset of first clinical signs.

Leveraging age at first clinical symptoms and the three domains of NSS (motor coordination, motor integration, and sensory integration), the prediction of treatment response in schizophrenia showed a good performance with an area-under-the-curve of 77% ± 8%, *p* = 1 × 10^−14^ (Fig. 3a). Mean F1-score was 0.65, specificity 0.73, sensitivity 0.64, and positive predictive value 0.66. Motor integration showed the highest absolute contribution with an average coefficient of 0.45, followed by sensory integration at 0.34, motor coordination at 0.31, and age at onset of clinical symptoms at −0.21. The distribution of treatment response according to NSS and age at onset of clinical symptoms is shown in Fig. 3b, where the high specificity of the high NSS/low age is illustrated by an upper left corner including only subjects with treatment resistance.

## Discussion

Most psychiatric disorders share a certain level of neurodevelopmental burden (Marín, 2016; Morris-Rosendahl & Crocq, 2020), whose quantification could help identify endophenotypes associated with various prognostic risks (Arango et al., 2018). In this cross-sectional study including subjects with schizophrenia and bipolar disorder, we found neurodevelopmental correlates of treatment response during the initial phase of treatment, which were also performant in predicting therapeutic response in schizophrenia. Transdiagnostically, in both schizophrenia and bipolar disorder, the presence of NSS was strongly associated with treatment resistance. An earlier onset of first subjective symptoms and first clinical signs were also strongly associated with resistance in schizophrenia. Age at first clinical signs and NSS markedly predicted resistance in schizophrenia.

Early developmental anomalies were significantly increased transdiagnostically compared to healthy controls, in particular for the presence of a history of obstetric complications, hyperactivity, dyslexia, epilepsy, and primary enuresis. They were not significantly greater in treatment-resistant groups than in the treatment-responsive groups. This might be due to a lack of power since there were missing data. Nevertheless, NSS were markedly higher, in treatment-resistant subjects compared to treatment-responsive, both in schizophrenia and bipolar disorder. These results are in line with previous case–control studies. High NSS were described in bipolar disorder and schizophrenia at relatively similar levels (Bora, Akgül, Ceylan, & Özerdem, 2018; Zhao et al., 2013). In mood disorders, it has been suggested that their presence may distinguish between bipolar and unipolar depression, translating the higher neurodevelopmental burden associated with bipolarity (Sagheer et al., 2018). In addition, NSS seem to convey a neurodevelopmental dimension linked to genetic risk, as offspring of subjects with schizophrenia or bipolar disease also present high NSS (Sugranyes et al., 2017). However, only a handful of studies focused on the relevance of NSS to predict treatment outcomes, while there is no literature on the subject, to our knowledge, in bipolar disease. In schizophrenia, some studies suggest a bidirectional influence of NSS and treatment-resistance: NSS may predict treatment-resistance (de Bartolomeis et al., 2018), while they may increase with time in treatment-resistant subjects (Lui et al., 2021). Our study thus further replicated NSS performance as a predictor of treatment-resistance in schizophrenia and suggests a transdiagnostic influence. Moreover, our analysis in the antipsychotic-naive subgroup found that NSS were already very significantly increased, in line with the literature on neuroleptic-naive subjects that found that NSS predated medication as a marker of trait (Krebs et al., 2000). We also found a correlation between NSS and extrapyramidal side-effects of medication, in line with the previous literature on NSS as marker of state and a possible influence of medication (Bachmann & Schröder, 2018). Nevertheless, the absence of difference in extrapyramidal side-effects between responders and resistant patients is a strong argument to be made in favor of NSS association with treatment resistance, independently of medication side-effects.

In bipolar disease, we also found higher NSS scores among treatment-resistant subjects, although this difference did not hold after correction for multiple testing, possibly due to lack of power.

Age at onset of disease is a well replicated risk factor of disease resistance, in schizophrenia and bipolar disorder (Joslyn et al., 2016; Smart et al., 2021). Classically, it is defined as the age at which the first symptoms of the disease appeared (Demjaha et al., 2017). However, in the clinical staging paradigm, early intervention studies have shown that non-specific distress, and symptoms such as anxiety and depression below diagnostic threshold occurred transdiagnostically and already represented a functional impairment that was relevant to the patient regardless of diagnostic criteria (Hasler, Moergeli, & Schnyder, 2004; McGorry, Hartmann, Spooner, & Nelson, 2018). For this reason, we also considered age at first subjective impairment in our study. Interestingly, in schizophrenia but not bipolar disease, subjects resistant to treatment complained of a functional impairment that occurred on average one year before the appearance of more specific psychotic clinical symptoms, and subjective impairment occurred in resistant subjects three years earlier than in responsive subjects, a difference that remained significant after correction for multiple testing. This was also the case for age at first clinical signs, which was two years lower in resistant subjects than in responsive ones. Our observation thus further argues in favor of a staging model incorporating early subjective distress as an important transnosographic information. Nevertheless, for prediction purposes, we chose to use the age at first clinical signs as it provides a more objective assessment of the disease.

Beyond neurodevelopmental markers of treatment response, longitudinal studies have found that higher disease-specific symptoms, male sex, lower education, quality of life and functioning, as well as a greater number of previous episodes, negative life events, and a poorer premorbid adjustment with more severe baseline psychopathology and a longer duration of untreated illness were associated with poorer outcomes (Carbon & Correll, 2014; Solmi et al., 2023). Here, we have also found that worse global functioning and higher general psychopathology were transdiagnostically associated with treatment resistance. In schizophrenia, there were higher positive, negative, and disorganization scores in treatment resistance than in response, and in bipolar disorder they were more depressive symptoms. However, in this cross-sectional setting, it cannot be assumed that these variables predate treatment-resistance, while such an assumption can tentatively be made for neurodevelopmental markers, considered to reflect early trajectory anomalies.

A strength of our study lies in the fact that it focused on distinguishing an excellent initial response (defined by a full recovery at six months) from treatment resistance in general (insufficient or no recovery), providing the means for early detection of non-responders and closer monitoring in order to reduce the delay to clozapine introduction in schizophrenia. Moreover, beyond group mean differences evidenced by significant *p* values, the non-parametric effect-size computations and the supervised machine-learning analysis showed that the differences in NSS and age at first clinical symptoms were directly relevant at the individual level. Several limitations can be discussed. First, our study is cross-sectional, and the characterization of resistance and all potentially explanatory variables was made retrospectively, albeit based on all available sources of information (prospectively documented clinical records, relatives, psychiatrists) allowing a comprehensive treatment history (types and duration) and the use of a global assessment scale. For bipolar disorders, the ALDA scale was used retrospectively. In search of more reliable developmental information, considering the participants were adults, we chose developmental variables easy to remember or found in the health booklet. But there was still a level of missing data for each variable, as indicated in the tables. However, there were enough subjects with NSS, age at onset, and treatment response information to allow prediction of resistance in schizophrenia (*N* = 197). As there was not, to our knowledge, any other independent cohort with these neurodevelopmental variables available, we used a cross-validation strategy to confirm their relevance in predicting treatment outcome. Also, despite the performant statistical prediction of our model, the cross-sectional design makes it a classification and a prospective study will be needed to check for longitudinal prediction. Longitudinal studies looking at NSS and age at onset in schizophrenia support our observations (Chan et al., 2021; Ferruccio et al., 2021). Finally, due to a smaller sample size, results in the bipolar disorder group were less conclusive (both in significance and effect-size) than what we found in schizophrenia, and supervised analysis could not be done.

In conclusion, NSS and age at first clinical signs appear to be strong predictors of treatment response in schizophrenia. A similar trend is observed in bipolar disorder but needs further replication. These transdiagnostic markers of a higher neurodevelopmental burden, accessible since the early stage of the disease, allow early detection of individuals at high risk of resistance. These results will help improve clinical practice by providing a means to identify patients who will require rapid treatment adaptation, such as clozapine introduction in schizophrenia. Prospective studies are further required to validate the predictive performance of these neurodevelopmental markers.
